# Clinical outcomes and safety of efgartigimod in Guillain–Barré syndrome: a retrospective observation study

**DOI:** 10.3389/fimmu.2026.1823319

**Published:** 2026-06-19

**Authors:** Yongli Tao, Ting Yang, Chenyang Jiang, Xue Wang, Liangliang Gu, Rui Zhang

**Affiliations:** 1 Department of Neurology, the First Affiliated Hospital of Zhengzhou University, Zhengzhou, China; 2 Department of Neurology, Nanyang Central Hospital, Nanyang, China

**Keywords:** retrospective observation study, efgartigimod, Guillain–Barre´ syndrome, outcomes, therapy

## Abstract

**Background:**

Guillain–Barré syndrome (GBS) is a severe acute autoimmune neuropathy with limited established therapeutic options. Efgartigimod, a human IgG antibody Fc fragment, can increase IgG degradation, which thus may be a promising therapeutic medicine for GBS. The aims of this study were to evaluate the clinical effectiveness and safety of efgartigimod in GBS patients.

**Methods:**

A retrospective study was conducted on GBS patients at the first affiliated hospital of Zhengzhou university and Nanyang central hospital from February 2024 to June 2025. The medical records of patients received IVIg, efgartigimod, or IVIg sequential efgartigimod (ISE) were reviewed. Disease severity was evaluated using GBS disability score (GBS-DS) and Inflammatory Neuropathy Cause and Treatment (INCAT) disability score at baseline, week1, week2, week3, week4, and the final follow-up. The primary outcome was the proportion of good improvement, defined as a reduction of at least 2 points in the GBS-DS score compared to the baseline. All adverse events occurred during the treatment period were documented.

**Results:**

52 patients were enrolled and received IVIg (n=20), efgartigimod (n=16), or ISE (n=16). Good improvement was higher in the efgartigimod group than IVIg group both at week 2 (37.5% vs. 0%, p<0.01) and the final visit (81.3% vs. 40.0%, p=0.02). Throughout follow-up, the proportion with GBS-DS ≤1 in the efgartigimod group was consistently higher compared to the other two groups. Although not statistically significant, the mean time to reach GBS-DS ≤1 was shorter in the efgartigimod group (2.6± 1.2 weeks) compared to the IVIg (3.1 ± 1.1 weeks), In the ISE group, the average time to achieve GBS-DS ≤ 1 was 2.8 ± 1.2 weeks. Regarding safety, the incidence of treatment-related adverse events (TEAEs) was lower in the efgartigimod group18.8% (3/16) than in the IVIg50.0% (10/20) and ISE 43.8% (7/16) groups.

**Conclusions:**

Based on the preliminary finding, the retrospective real-world data suggesting a potential signal of benefit for efgartigimod in GBS, with no new safety concerns identified. However, this study cannot establish comparative efficacy given the methodological limitations, and large-scale well-controlled head-to-head trials remain imperative.

## 1 Introduction

Guillain–Barré syndrome (GBS) is an immune-mediated polyneuropathy, with an annual global incidence of approximately 100,000 cases (1, 2). It typically manifests as an acute, progress, symmetrical, usually ascending flaccid paralysis accompanied by weak or absent tendon reflexes (3). Although the specific details of the pathogenesis of Guillain–Barré syndrome (GBS) remain to be fully elucidated, recent research focusing on the phenomenon of post-infection molecular mimicry has revealed the involvement of IgG autoantibodies and innate immune effectors in the disease (4). Currently, intravenous immunoglobulin G (IVIg) and therapeutic plasma exchange (TPE) are the main immunotherapies recommended by guidelines for GBS (5–7). However, IVIg and TPE are not always available, especially given the growing global shortage of blood products. And some patients treated with IVIg had serious side effects, such as thrombosis, aseptic meningitis, renal insufficiency, elevated neutrophils, and so on (8–10). Notably, despite the application of conventional immunotherapeutic approaches, around 3–7% of patients die, 16% of severe cases admitted to the intensive care unit (ICU) remain unable to walk independently at one year, and about 30% still experience chronic pain (11). Therefore, there is an urgent need for effective, rapidly acting, and well-tolerated therapeutic drugs for GBS patients, independent of blood donations.

Efgartigimod is the first blocks the neonatal Fc receptor, which is involved in IgG recycling, can increase IgG degradation and depletion and thus reduction in pathogenic IgG antibodies (12). In the Phase I and II trials of efgartigimod in generalized myasthenia gravis, the efgartigimod significantly reduced IgG levels and just with mostly mild and self-limiting adverse events, which provide a basis for treating IgG autoantibody-mediated diseases (13, 14). Besides treating myasthenia gravis (15), there are several studies reported on other autoimmune diseases, such as chronic inflammatory demyelinating polyradiculoneuropathy, primary immune thrombocytopenia, autoimmune encephalitis, pemphigus vulgaris, and foliaceus (16–18). And previous case reports and small sample study have shown that efgartigimod seems effective, safe, and may shorten the course of GBS (1, 12, 19–22).

The primary objective of this study was to describe the clinical outcomes and safety profile of efgartigimod in patients with GBS, based on a retrospective, descriptive analysis of data from our institutions.

## 2 Materials and methods

### 2.1 Study population

This was a dual-center retrospective observational cohort study conducted in the First Affiliated Hospital of Zhengzhou University and Nanyang Central Hospital between February 2024 and June 2025. We collected individuals who were over 18years old, fulfilled the Brighton diagnostic criteria (23) for GBS, and GBS Disability Scale (GBS-DS) ≤ 5 at screening. We enrolled a cohort of GBS patients treated with IVIg, efgartigimod(to mitigate the risks associated with standard therapies, such as severe intolerance to IVIg or contraindications to IVIg, patient preference, IVIg accessibility at admission), or IVIg sequential efgartigimod (ISE). The cohort as a part of the Registration and Research on Neuroimmune Diseases, which was approved by the institutional review boards of the first affiliated hospital of Zhengzhou university (EC No:2021-KY-0822-002).

### 2.2 Information collection

Basic information and clinical characteristics of the included patients were collected, including demographic data, previous history, symptoms, auxiliary examination findings, GBS classification, treatment profiles throughout the disease course, neurological status, and the time from treatment to stabilization of the condition.

### 2.3 Treatment and follow-up

The treatment protocols were as follows: 1) IVIg Group: Patients received intravenous immunoglobulin G at a standard dose of 0.4 g/kg per day for five consecutive days. 2) Efgartigimod Group: Patients received efgartigimod at a dose of 10 mg/kg via intravenous infusion over 1 hour. The number of infusions (ranging from 1 to 5) was determined based on the individual’s symptomatic response and patient preference.3) IVIg sequential efgartigimod (ISE) Group: Patients first received the full course of IVIg treatment (0.4 g/kg/day for 5 days). Following this, they subsequently switched to efgartigimod therapy, with the dosage and administration schedule identical to that used in the efgartigimod-alone group.

In this study, disease severity was evaluated using the Guillain–Barré syndrome disability score (GBS-DS) and the Inflammatory Neuropathy Cause and Treatment (INCAT) disability score. These scores were extracted from the inpatient and outpatient departments medical records at predetermined time points: baseline and at 1, 2, 3, and 4 weeks post-treatment. To assess the long-term prognosis of patients across different treatment regimens, a final follow-up was conducted in August 2025. Data from the final follow-up was obtained through interviews (face-to-face or via video) conducted with patients’ informed consent.

### 2.4 Outcomes

The efficacy outcomes comprised: (1) good improvement: defined as a reduction of at least 2 points in the GBS-DS score compared to the baseline at each follow-up visit. (2) proportion of GBS-DS ≤ 1 at each follow-up time point. (3) time to achieve GBS-DS ≤1 in each treatment group. (4) absolute reduction in GBS-DS score from baseline at all timepoints. (5) change in INCAT score relative to baseline. (6) disease duration in different treatment groups. The safety outcome was any adverse events, which occurred during the treatment period were recorded. The treatment-related adverse events (TEAEs) mainly included the following: infection (respiratory, urinary system, etc.), venous thrombosis, electrolyte imbalance, herpes, hypoalbuminemia, liver dysfunction, anemia.

### 2.5 Statistical analysis

Statistical analyses were performed using R (version 4.4.1). The variables of normally distributed were expressed as mean ± standard deviation (Mean ± SD), and comparison between three groups were analyzed using a t-test; Variables that were not normally distributed were expressed as the interquartile range (IQR) and Mann-Whitney *U* tests were used to compare groups of differences clinical characteristics and outcomes. The categorical data were expressed in frequency (percentage), and the comparison between groups were conducted using chi-square test or Fisher’s exact test. The GBS-DS scores of different groups and time points were evaluated using generalized estimation equations. P<0.05 was considered statistically significant, and all tests were two-side.

## 3 Results

### 3.1 Baseline characteristics of the study population

**Figure 1:**
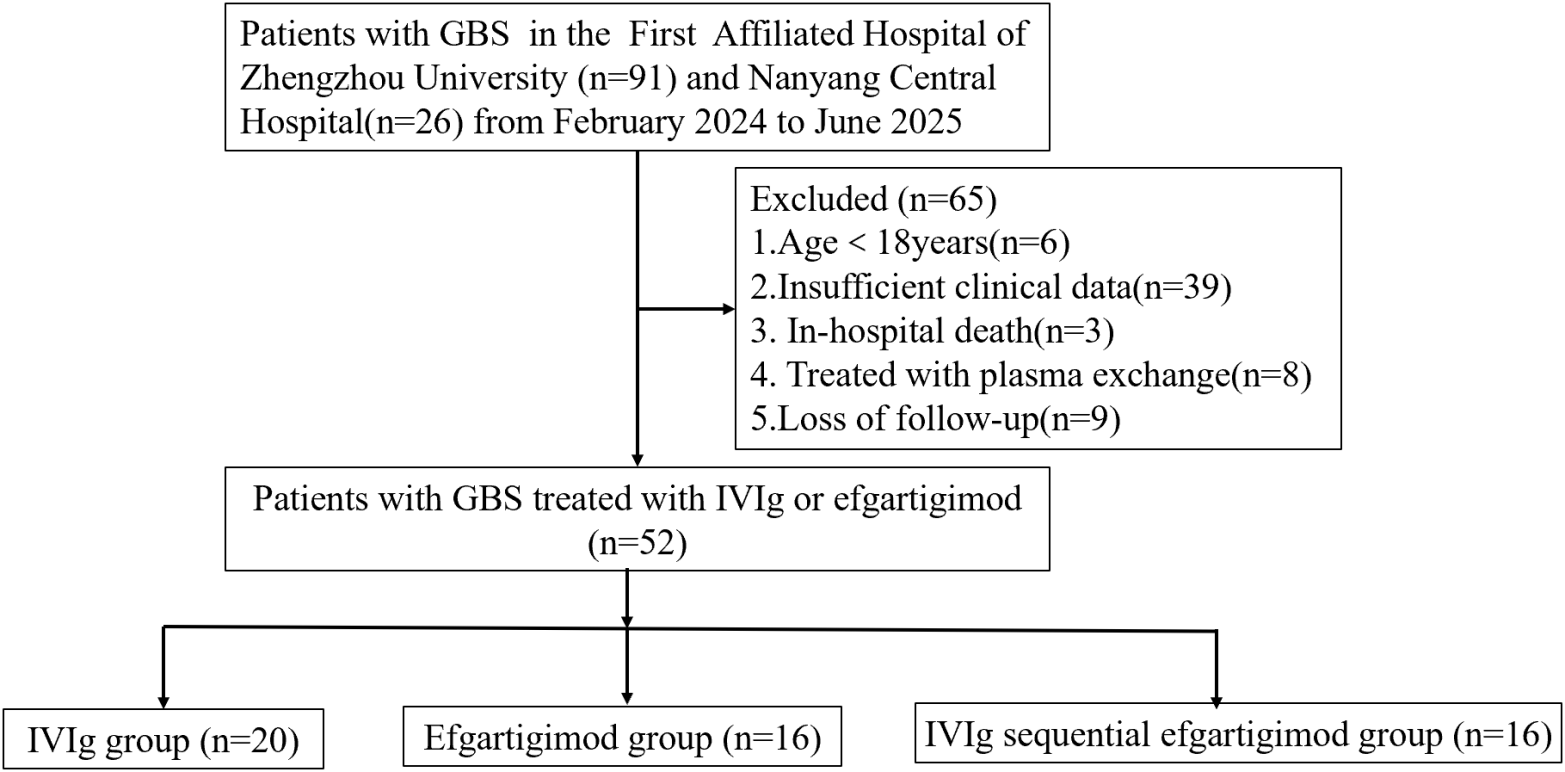
Flow chat of patients enrolled. GBS, Guillain–Barré syndrome; IVIg, intravenous immunoglobulin G.

From Feb 2024 to Jun 2025, a total of 52 patients with GBS were enrolled in the study, including 20 patients treated with IVIg,16 patients treated with efgartigimod, and 16 patients in the ISE group. A summary of the patient selection process is presented in **Figure 1**. The baseline characteristics of different groups were shown in the **Table 1**. No significant differences were found among three groups in terms of age, gender, history of diabetes, infection history before onset, disease course before admission, follow-up time, cerebrospinal fluid protein content, INCAT, GBS-DS, disease subtype, and whether mechanical ventilation was used during hospitalization(p>0.05).

**Table 1:** The baseline characteristic between difference treatment groups.

Characteristics	IVIg (N = 20)	Efgartigimod (N = 16)	ISE (N = 16)	P value
Age, years, Mean ± SD	50.1 ± 16.7	58.1 ± 13.7	48.6 ± 13.3	0.15
Female, n, (%)	9 (45.0)	6 (37.5)	8 (50.0)	0.77
History of diabetes, n (%)	2 (10.0)	3 (20.0)	1 (6.3)	0.56
Infection history, n (%)	8 (40.0)	3 (18.8)	7 (43.8)	0.27
Per-admission course, IQR, d	6.0 (3.0-11.0)	6.5 (4.0-11.3)	3.5 (3.0-8.0)	0.30
Follow-up duration, d, Mean ± SD	240.5 ± 112.6	218.50± 135.7	261.2 ± 136.8	0.64
Protein of CSF, mg/dl, IQR	576.7 (463.1-1227.7)	519.4 (373.0-791.2)	571.1 (291.7-968.8)	0.64
INCAT score, Mean ± SD	5.9 ± 3.1	5.9 ± 3.1	5.9 ± 3.3	1.00
GBS-DS score, IQR	3.0 (3.0-4.0)	3.0 (2.0-4.0)	3.50(3.0-4.0)	0.48
Disease subtype, n (%)				0.26
AIDP	8 (40.0)	5 (31.3)	7 (43.8)	
AMAN	8 (40.0)	10 (62.5)	4 (25.0)	
AMSAN	3 (15.0)	0 (0.0)	4 (25.0)	
MFS	1 (5.0)	1 (6.3)	1 (6.3)	
Ventilator support, n (%)	3 (15.0)	0 (0.0)	3 (18.8)	0.29

IVIg, intravenous immunoglobulin G; ISE, intravenous immunoglobulin G sequential efgartigimod; IQR, interquartile range; CSF, cerebrospinal fluid; INCAT, inflammatory neuropathy cause and treatment scale; GBS-DS, Guillain–Barré syndrome disability score; AIDP, acute inflammatory demyelinating polyneuropathies; AMAN, acute motor axonal neuropathy; AMSAN, acute motor⁃sensory axonal neuropathy; MFS, Miller⁃Fisher syndrome.

### 3.2 Good improvement in GBS-DS scores among different groups

The **Table 2** results showed that there were differences in the good improvement among the three groups at week 2 (0 vs.37.5%vs.6.3%), and the final follow-up (40.0%vs.81.3%vs.75.0%). Further pairwise comparisons revealed that at these two time points, the improvement in the efgartigimod group was higher than that in the IVIg group, and the difference was statistically significant[(37.5% vs.0, p<0.01) and(81.3% vs. 40.0%, p=0.02)].

**Table 2:** Differences analysis of improvement in GBS-DS ≥2 points in different groups.

Times	IVIg (n=20)	Efgartigimod (n=16)	ISE (n=16)	P value
Week1				0.28
< 2	20(100.0)	14(87.5)	15(93.8)	
≥2	0(0)	2(12.5)	1(6.3)	
Week2				<.01
< 2	20(100.0)	10(62.5)	15(93.8)	
≥2	0(0)	6(37.5)*	1(6.3)	
Week3				0.30
< 2	14(70.0)	9(56.3)	13(81.3)	
≥2	6(30.0)	7(43.8)	3(18.8)	
Week4				0.50
< 2	12(60.0)	7(43.8)	10(62.5)	
≥2	8(40.0)	9(56.3)	6(37.5)	
The Final				0.02
< 2	12(60.0)	3(18.8)	4(25.0)	
≥2	8(40.0)	13(81.3)*	12(75.0)	

IVIg, intravenous immunoglobulin G; ISE, intravenous immunoglobulin G sequential efgartigimod.

*IVIg group as the reference, p<0.05.

Differences in treatment timing may confound the observed effects. Therefore, we included “time from symptom onset to treatment initiation (i.e., pre-admission disease duration in **Table 1**)” as a key variable in our analysis. Baseline intergroup comparisons showed no statistically significant difference in treatment timing among the three groups (P = 0.300; **Table 1**), indicating that treatment timing was balanced across the different treatment groups. To further explore the relationship between treatment timing and outcomes, we analyzed the correlation between pre-admission disease duration and GBS-DS scores at each time point. The results showed that treatment timing was not significantly correlated with improvements in GBS-DS at any follow-up time point (Spearman correlation coefficients r = -0.078, -0.057, -0.013, 0.173, and -0.001, respectively; all P > 0.05), suggesting that treatment timing did not significantly affect clinical outcome improvements in our study population.

We categorized changes in GBS-DS from baseline as: worsened, 0 (no change), 1-point improvement, or ≥2-point improvement. While overall improvement patterns were similar across the three treatment groups, the efgartigimod group consistently demonstrated a higher proportion of patients achieving ≥2-point GBS-DS improvement at every follow-up timepoint compared to both the IVIg and ISE groups (**Supplementary Table 1**). One efgartigimod-treated patient experienced clinical worsening related to traumatic intracranial hemorrhage, while a patient in the ISE group showed temporary symptom aggravation during IVIg infusion that ameliorated after switching to efgartigimod. This observation also suggests that efgartigimod may facilitate greater magnitude of symptomatic improvement in GBS patients.

The results of univariate logistic regression for improvement of GBS-DS score at the last follow-up are presented in **Supplementary Table 2**. Baseline INCAT and GBS-DS were both influencing factors for the improvement of GBS-DS score. Specifically, compared with the IVIG group, the odds ratio (OR) of improvement in GBS-DS score at the last follow-up in the efgartigimod group was 5.30 times that of the IVIg group (OR = 5.3, 95% CI: 1.1- 24.6, P = 0.03). Each 1-point increase in baseline INCAT was associated with a 25% greater likelihood of improvement (OR = 1.3, 95% CI: 1.0-1.5, P = 0.03), and each 1-point increase in baseline GBS-DS (OR = 1.9, 95% CI: 1.0-3.7, P = 0.05), the improvement advantage increased by 93%. Age, gender, history of diabetes, infection history before onset, per-admission course, cerebrospinal fluid protein, and mechanical ventilation were not significantly associated with score improvement (P > 0.05).

### 3.3 Efficacy of different treatments

By comparing the proportion of patients with GBS-DS scores ≤1 across the treatment groups at week 1, week 2, week 3, week 4, and the final visit, we observed that at week 1, there was 1 patient (5.0%) in the IVIg group, 2 patients (12.50%) in the efgartigimod group, and 1 patient (6.3%) in the ISE group with GBS-DS scores ≤1 (p=0.82). By week 4, the proportion increased to 9 patients (45.0%) in the IVIg group, 10 patients (62.5%) in the efgartigimod group, and 6 patients (37.5%) in the ISE group. Detailed results for other time points are provided in **Table 3**. Throughout the weekly and the final follow-ups, the proportion of patients with GBS-DS ≤1 in the efgartigimod group was consistently higher than in the other two groups, although these differences did not reach statistical significance (**Table 3**). We further analyzed the GBS-DS scores before and after treatment across the groups. The results indicated that only the time effect was statistically significant (P<0.01), with no significant group effect or interaction observed (**Supplementary Table 3**).

**Table 3:** Differential analysis of the proportion of GBS-DS score ≤ 1 after treatment in these groups.

Times	IVIg (n=20)	Efgartigimod (n= 16)	ISE (n=16)	P value
Week1	1(5.0)	2(12.5)	1(6.3)	0.82
Week2	2(10.0)	5(31.3)	2(12.5)	0.27
Week3	5(25.0)	7(43.8)	4(25.0)	0.49
Week4	9(45.0)	10(62.5)	6(37.5)	0.35
The Final	11(55.0)	13(81.3)	12(75.0)	0.24

IVIg, intravenous immunoglobulin G; ISE, intravenous immunoglobulin G sequential efgartigimod.

### 3.4 Time to achieve GBS-DS ≤ 1 in different treatment groups

The **Table 4** presents the time required for different groups to achieve a GBS-DS score of ≤1 after treatment. The time for the efgartigimod group to achieve GBS-DS ≤ 1 was 2.6 ± 1.2 weeks, which was shorter than that of the IVIg group (3.1 ± 1.1weeks) and the ISE group (2.8 ± 1.2weeks). However, no statistical difference was observed (p=0.62).

**Table 4:** Differential analysis of the time required for different groups to achieve GBS-DS score ≤ 1 after treatment.

Variables	IVIg (n=9)	Efgartigimod (n=10)	ISE (n=6)	P value*
Time, week	3.1 ± 1.1	2.6 ± 1.2	2.8 ± 1.2	0.62

IVIg, intravenous immunoglobulin G; ISE, intravenous immunoglobulin G sequential efgartigimod.

* the ISE group as the reference.

### 3.5 Trend of GBS-DS and INCAT changes in the three groups

**Figure 2:**
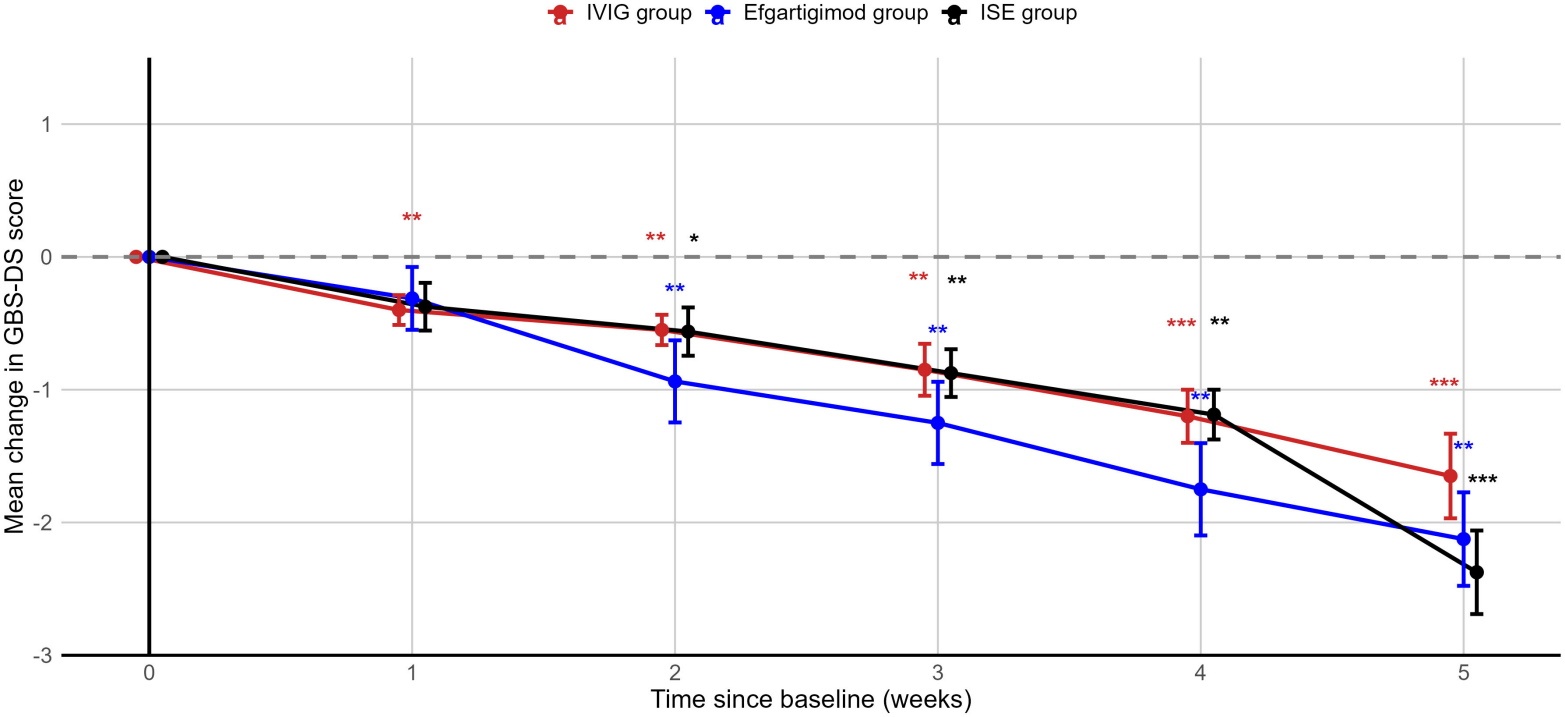
The trend of GBS-DS scores at each visit point among different groups. IVIg, intravenous immunoglobulin G; ISE, intravenous immunoglobulin G sequential efgartigimod. *p < 0.05, **p < 0.01, ***p < 0.001.

**Figure 3:**
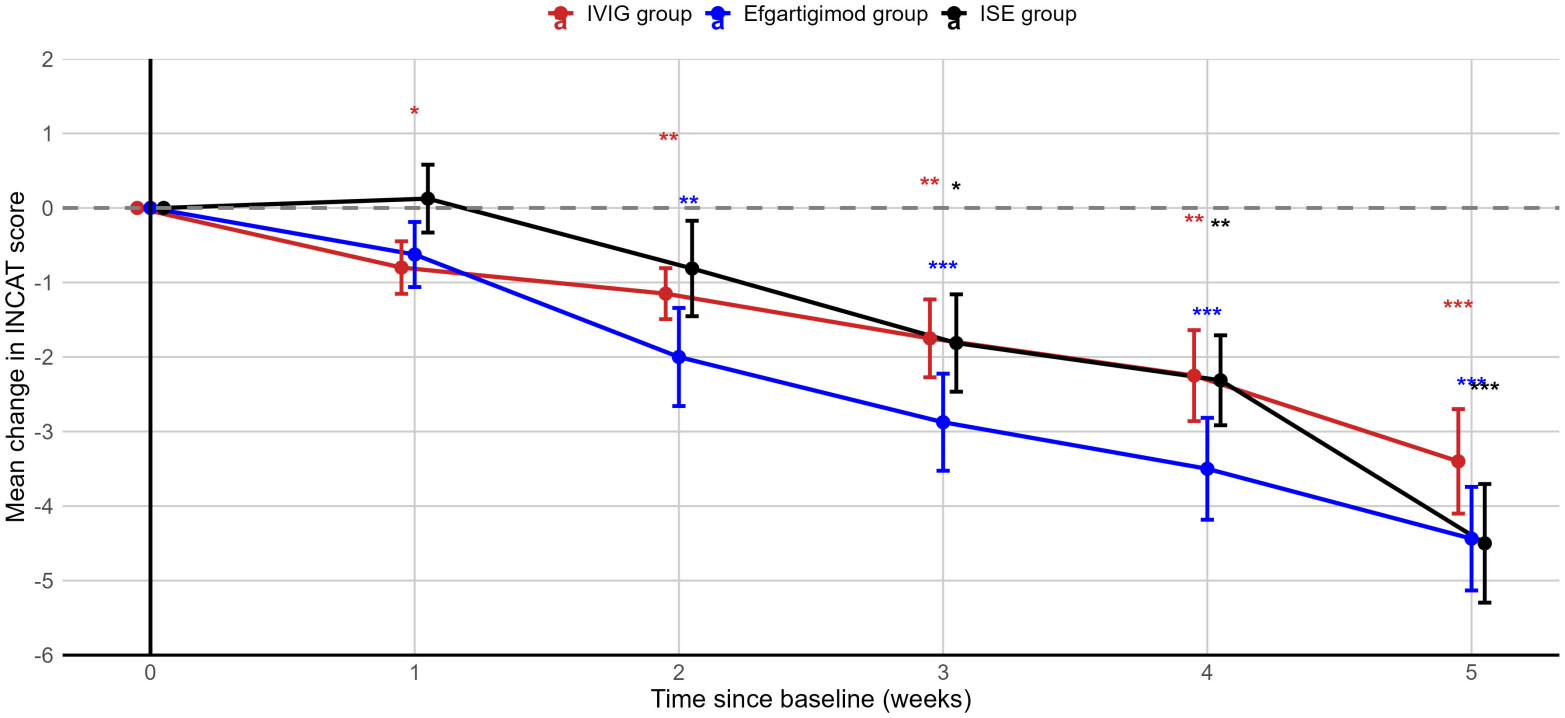
The trend of INCAT scores at each visit point among different groups. IVIg, intravenous immunoglobulin G; ISE, intravenous immunoglobulin G sequential efgartigimod. *p < 0.05, **p < 0.01, ***p < 0.001.

By plotting the trend of GBS-DS score changes in different groups, the horizontal axis represents time (such as baseline, week1, week2, week3, week4, and the final visit), and the vertical axis represents the change in the mean score relative to baseline (before treatment). The results showed that for inter group comparison, the difference in GBS-DS gradually increased in different groups after treatment, and the efgartigimod group showed a faster decline trend, but no differences were found in the changes in scores at various time points among the three groups; For intra group comparison, compared with baseline, all three groups showed significant decreases starting from Week 2, and the differences were statistically significant, as shown in **Figure 2**. The changes of INCAT scores were consistent with GBS-DS (**Figure 3**).

### 3.6 Disease duration comparison across treatment groups

Two patients in the IVIg group who died at 2 months post-onset were excluded from this analysis. We observed significant differences in median time to disease stabilization among the treatment groups (IVIg: 18.0days; efgartigimod: 13.5days; ISE: 26days; P = 0.03). Although pairwise comparisons did not reach statistical significance, the efgartigimod group demonstrated a clinically relevant reduction in disease duration compared to both IVIg monotherapy and switched therapy (**Table 5**).

**Table 5:** The time of stable disease after treatment among patients in different groups.

Variables	IVIg (n=18)	Efgartigimod (n=16)	ISE (n=16)	P value
Time to stabilization, d, (IQR)	18.0(12.3-41.3)	13.5(6.3-23.8)	26.0(19.0-45.3)	0.03

IVIg, intravenous immunoglobulin G; ISE, intravenous immunoglobulin G sequential efgartigimod; d, days; IQR, Interquartile range.

### 3.7 Treatment-related adverse events

Among the 52 patients, 23patients occurred TEAEs. In the IVIg group, 12 patients (60%) occurred TEAEs, consisting of 6venous thrombosis, 3electrolyte imbalance, 6 pulmonary infection, 1 hypoalbuminemia, 1 herpes, 1 anemia. In the efgartigimod group, 3patients (15%) experienced TEAEs, including 2venous thrombosis and 1electrolyte imbalance. In the ISE group, 7 patients (43.8%) experienced TEAEs, which included 1venous thrombosis, 1electrolyte imbalance, 7 infection (6 pulmonary infection and 1urinary system infection), 1herpes, 2 hypoalbuminemia, 1 anemia, and 5liver dysfunction. The IVIg group reported two patient died two months after onset of GBS. One patient in the efgartigimod group experienced death related to traumatic intracranial hemorrhage.

## 4 Discussion

As a retrospective observational cohort study rather than a controlled clinical trial, this work provides a novel, real−world descriptive analysis of efgartigimod in patients with GBS, including its efficacy and tolerability profile. However, given the numerous methodological limitations inherent to this design, the study cannot establish comparative efficacy among the three treatment groups. The observed clinical outcomes, along with the safety data, should be regarded as purely hypothesis−generating. These preliminary observations require validation in appropriately powered, prospective, randomized controlled trials. GBS originates from a dysregulated immune response, commonly instigated by preceding infections with pathogens such as Campylobacter jejuni, cytomegalovirus, or Epstein-Barr virus (24). The ensuing autoimmune reaction is misdirected toward peripheral nerve structures, culminating in a spectrum of pathological effects: demyelination, inflammatory neuropathy, and axonal degeneration (25). According to current treatment guidelines, IVIg and TPE stand as the exclusively recognized immunotherapeutic interventions that demonstrate efficacy in shortening the recovery course of GBS (7, 26). However, both treatments relied on voluntary blood donations. Efgartigimod is a human IgG1–derived Fc fragment that has been modified using ABDEG technology to increase its affinity for FcRn at both physiological and acidic pH. And efgartigimod has a more effective and rapid impact on clearing IgG in cynomolgus monkeys compared to standard IVIg therapy (14).

Previous case reports and small series have suggested a potential clinical benefit of efgartigimod in patients with GBS, providing the rationale for this retrospective cohort study (1, 12, 19). In the present analysis, patients in the efgartigimod group showed a statistically significant improvement in GBS−DS scores at both week 2 and the last follow−up, suggesting that early improvement may be achieved and maintained. In the sequential treatment (ISE) group, which comprised patients with an inadequate response to initial IVIg, a numerically higher proportion achieved a ≥2−point improvement in GBS−DS at the final follow−up after the switch to efgartigimod. However, owing to the inherent indication bias, these data are descriptive only and cannot be directly compared with the first−line treatment groups, but they may still inform the design of future prospective trials specifically targeting IVIg-refractory patients. Regarding the endpoint of GBS−DS ≤ 1, no statistically significant difference was observed between the efgartigimod group and the other two groups at any follow−up time point. The proportion of patients reaching this endpoint was numerically higher, and the time to achieve it numerically shorter in the efgartigimod group. No new safety concerns were identified, and efgartigimod was generally well tolerated across the treatment groups. Collectively, these findings are hypothesis−generating and should not be taken as evidence of comparative efficacy. They suggest that efgartigimod may warrant further investigation as a potential treatment option for GBS, including in settings of IVIg shortage or contraindication, and underscore the need for well−designed, adequately powered, prospective randomized trials to test these hypotheses.

For the TEAEs, the efgartigimod group has the lowest incidence of TEAEs. Although three groups had venous thrombosis patients, the IVIg group was higher than the other groups, which is consistent with previous research findings (27). Moreover, in the IVIg group and ISE group, the incidence of pulmonary infection was higher, which might be related to the longer hospital stay and the use of ventilator-assisted ventilation. Furthermore, in the ISE group, all patients showed unsatisfactory improvement in their symptoms after IVIg treatment, the clinicians decided to administer efgartigimod sequentially. This group had a higher incidence of liver function abnormalities, which might be related to the fact that the patients were taking multiple medications simultaneously. Our results suggest that efgartigimod not only exerts rapid clinical effects in GBS, potentially preventing disease progression when administered early, but is also associated with lower complication rates and comparable cost-effectiveness (28).

However, our study has certain limitations. First, as a retrospective study, this work has several inherent limitations, including a small sample size, non−randomized treatment allocation, and baseline imbalances between the groups. These limitations underline that the findings are preliminary and must be interpreted with considerable caution. Second, because anti-ganglioside antibody testing was not performed in the majority of patients, this precluded any analysis of the relationship between antibody status and clinical outcomes. Third, owing to the small overall sample size, any subgroup analysis by electrophysiological subtype would have had insufficient statistical power and could have produced unstable or misleading results. Formal statistical comparisons of treatment response across subtypes were therefore not performed. Fourth, cerebrospinal fluid protein levels reported in this study were relatively low, because we just collected cerebrospinal fluid protein at baseline, which may have occurred relatively early after disease onset.

## 5 Conclusion

In this small, retrospective cohort study, efgartigimod was associated with numerically favorable clinical trajectories and an acceptable safety profile in GBS patients. However, because of the inherent limitations of the study design, no conclusions regarding comparative efficacy can be drawn. These observations are hypothesis−generating only and underscore the need for adequately powered, prospective randomized controlled trials to rigorously evaluate the potential therapeutic role of efgartigimod in GBS.

## Data Availability

The datasets generated and analyzed during the current study are not publicly available because the data are currently being utilized for additional analysis, but are available from the corresponding author on reasonable request.
