# Supplementary material for: Clinical outcomes and safety of efgartigimod in Guillain–Barré syndrome: a retrospective observation study
Source: Front Immunol. 2026 Jun 19;17:1823319. doi: 10.3389/fimmu.2026.1823319 (PMC13374791; doi:10.3389/fimmu.2026.1823319)
Supplement: Supplementary file 1 [file Table1.docx]

Table S1. Differential analysis of changes in GBS-DS scores in different groups

| Times | IVIg  (n =20) | Efgartigimod  (n =16) | ISE  (n =16) | **P Value** |
| --- | --- | --- | --- | --- |
| Week1 |  |  |  | 0.91 |
| Worsen | 0（0） | 1（6.3） | 1（6.3） |  |
| No change | 12（60.0） | 10（62.5） | 9（56.3） |  |
| 1 | 8（40.0） | 3（18.8） | 5（31.3） |  |
| ≥2 | 0（0.0） | 2（12.5） | 1（6.3） |  |
| Week2 |  |  |  | 0.32 |
| Worsen | 0（0） | 1（6.3） | 1（6.3） |  |
| No change | 9（45.0） | 5（31.3） | 6（37.5） |  |
| 1 | 11（55.0） | 4（25.0） | 8（50.0） |  |
| ≥2 | 0（0） | 6（37.5） | 1（6.3） |  |
| Week3 |  |  |  | 0.34 |
| Worsen | 0（0） | 1（6.3） | 0（0） |  |
| No change | 9（45.0） | 2（12.5） | 5（31.3） |  |
| 1 | 5（25.0） | 6（37.5） | 8（50.0） |  |
| ≥2 | 6（30.0） | 7（43.8） | 3（18.8） |  |
| Week4 |  |  |  | 0.40 |
| Worsen | 0（0） | 1（6.3） | 0（0） |  |
| No change | 5（25.0） | 0（0） | 3（18.8） |  |
| 1 | 7（35.0） | 6（37.5） | 7（43.8） |  |
| ≥2 | 8（40.0） | 9（56.3） | 6（37.5） |  |
| The Final |  |  |  | 0.75 |
| Worsened | 3（15.0） | 1（6.3） | 0（0） |  |
| No change | 0（0） | 0（0） | 0（0） |  |
| 1 | 9（45.0） | 2（12.5） | 4（25.0） |  |
| ≥2 | 8（40.0） | 13（81.3） | 12（75.0） |  |

Abbreviation: IVIg, intravenous immunoglobulin G. ISE: intravenous immunoglobulin G sequential efgartigimod.
