# Supplementary material for: Clinical outcomes and safety of efgartigimod in Guillain–Barré syndrome: a retrospective observation study
Source: Front Immunol. 2026 Jun 19;17:1823319. doi: 10.3389/fimmu.2026.1823319 (PMC13374791; doi:10.3389/fimmu.2026.1823319)
Supplement: Supplementary file 2 [file Table2.docx]

TableS2. Univariate Logistic regression of factors associated with good improvement.

| Variables | OR (95%CI) | P Value |
| --- | --- | --- |
| Age, years | 1.0 (0.9 -1.0) | 0.36 |
| Sex |  |  |
| Male | 1.0 (Reference) |  |
| Female | 0.7 (0.2-2.2) | 0.54 |
| History of diabetes |  |  |
| No | 1.00 (Reference) |  |
| Yes | 0.5 (0.1-2.7) | 0.41 |
| Infection history |  |  |
| No | 1.0 (Reference) |  |
| Yes | 1.6 (0.5-5.6) | 0.45 |
| Per-admission course, d | 1.0 (0.9-1.1) | 0.64 |
| Protein of CSF, mg/dl | 1.00 (1.0-1.0) | 0.81 |
| INCAT, score | 1.3 (1.0-1.5) | 0.03 |
| GBS-DS, score | 1.9 (1.0 -3.7) | 0.05 |
| Disease subtype |  |  |
| AIDP | 1.0 (Reference) |  |
| AMAN | 1.8 (0.5-6.5) | 0.38 |
| AMSAN | 1.7 (0.3-10.8) | 0.59 |
| MFS | 0.33 (0.1-4.3) | 0.40 |
| Ventilator support |  |  |
| No | 1.0 (Reference) |  |
| Yes | 1.1 (0.2-6.5) | 0.94 |
| Groups |  |  |
| IVIg | 1.0 (Reference) |  |
| Efgartigimod | 5.3 (1.1-24.6) | 0.03 |
| ISE | 3.7 (0.9-15.4) | 0.08 |

Abbreviation: CSF, cerebrospinal fluid; INCAT, inflammatory neuropathy cause and treatment scale; GBS-DS, Guillain–Barré syndrome disability score; AIDP, acute inflammatory demyelinating polyneuropathies; AMAN, acute motor axonal neuropathy; AMSAN, acute motor⁃sensory axonal neuropathy; MFS, Miller⁃Fisher syndrome; IVIg, intravenous immunoglobulin G; ISE, intravenous immunoglobulin G sequential efgartigimod.
