# Supplementary material for: Clinical outcomes and safety of efgartigimod in Guillain–Barré syndrome: a retrospective observation study
Source: Front Immunol. 2026 Jun 19;17:1823319. doi: 10.3389/fimmu.2026.1823319 (PMC13374791; doi:10.3389/fimmu.2026.1823319)
Supplement: Supplementary file 3 [file Table3.docx]

Table S3. GBS disability score before and after treatment in different groups

| Groups | Baseline | Week1 | Week2 | Week3 | Week4 | The Final |  |
| --- | --- | --- | --- | --- | --- | --- | --- |
| IVIg | 3.4±1.0 | 3.0±1.2 | 2.9±1.2 | 2.6±1.3 | 2.2±1.5 | 1.8±1.8 |  |
| Efgartigimod | | 3.1±0.9 | 2.8±1.1 | 2.1±1.0 | 1.8±1.1 | 1.3±1.1 | 0.9±1.1 |
| ISE | 3.5±1.0 | 3.1±1.2 | 2.9±1.3 | 2.6±1.4 | 2.3±1.5 | 1.1±0.8 |  |
| F | 0.87 | 0.43 | 2.38 | 2.05 | 2.62 | 1.88 |  |
| P Value | 0.42 | 0.65 | 0.10 | 0.14 | 0.08 | 0.16 |  |
| Groups | Z=3.63, P=0.16 | | | | |  |  |
| Times | Z=46.68, P<.01 | | | | |  |  |
| Groups* Times | Z=10.11, P=0.43 | | | | |  |  |

Abbreviation: IVIg, intravenous immunoglobulin G. ISE: intravenous immunoglobulin G sequential efgartigimod.
